# SARS-CoV-2 variants and mutational patterns: relationship with risk of ventilator-associated pneumonia in critically ill COVID-19 patients in the era of dexamethasone

**DOI:** 10.1038/s41598-023-33639-5

**Published:** 2023-04-24

**Authors:** Keyvan Razazi, Anissa Martins Bexiga, Romain Arrestier, Bastien Peiffer, Guillaume Voiriot, Charles-Edouard Luyt, Tomas Urbina, Julien Mayaux, Tài Pham, Damien Roux, Raphael Bellaiche, Zakaria AIt Hamou, Stéphane Gaudry, Elie Azoulay, Armand Mekontso Dessap, Christophe Rodriguez, Jean-Michel Pawlotsky, Slim Fourati, Nicolas de Prost

**Affiliations:** 1 Médecine Intensive Réanimation, Hôpitaux Universitaires Henri Mondor, Assistance Publique-Hôpitaux de Paris (AP-HP), 51, Av de Lattre de Tassigny, 94000 Créteil Cedex, France; 2 Groupe de Recherche Clinique CARMAS, Université Paris-Est-Créteil (UPEC), Créteil, France; 3 Université Paris-Est-Créteil (UPEC), Créteil, France; 4 DMU Medecine, Hôpitaux Universitaires Henri Mondor, Assistance Publique-Hôpitaux de Paris (AP-HP), Créteil, France; 5 Médecine Intensive Réanimation, Hôpital Tenon, Assistance Publique-Hôpitaux de Paris, Paris, France; 6 Sorbonne Université, Assistance Publique-Hôpitaux de Paris, Hôpital Pitié-Salpêtrière, Médecine Intensive Réanimation, and INSERM UMRS_1166-iCAN, Institute of Cardiometabolism and Nutrition, Paris, France; 7 Sorbonne Université, Assistance Publique-Hôpitaux de Paris, Hôpital Saint-Antoine, Médecine Intensive Réanimation, 75571 Paris Cedex 12, France; 8 Sorbonne Université, Assistance Publique-Hôpitaux de Paris, Hôpital Pitié-Salpêtrière, Médecine Intensive Réanimation, Paris, France; 9 Service de Médecine Intensive-Réanimation, Assistance Publique-Hôpitaux de Paris, Hôpital de Bicêtre, DMU 4 CORREVE Maladies du Cœur et des Vaisseaux, FHU Sepsis, Le Kremlin-Bicêtre, France; 10 Université Paris-Saclay, UVSQ, Univ. Paris-Sud, Inserm U1018, Equipe d’Epidémiologie Respiratoire Intégrative, CESP, 94807 Villejuif, France; 11 Université de Paris, APHP, Hôpital Louis Mourier, DMU ESPRIT, Service de Médecine Intensive Réanimation, Colombes, France; 12 Department of Immunology, Infectiology and Hematology, INSERM U1151, CNRS UMR 8253, Institut Necker-Enfants Malades (INEM), Colombes, Paris, France; 13 Département d’Anesthésie Réanimations Chirurgicales, Hôpitaux Universitaires Henri Mondor, Assistance Publique-Hôpitaux de Paris, Créteil, France; 14 Médecine Intensive Réanimation, Hôpital Cochin, Assistance Publique-Hôpitaux de Paris, Paris, France; 15 Service de Réanimation, Hôpital Avicenne, Assistance Publique-Hôpitaux de Paris, Bobigny, France; 16 Médecine Intensive Réanimation, Hôpital Saint-Louis, Assistance Publique-Hôpitaux de Paris, Paris, France; 17 Department of Virology, Hôpitaux Universitaires Henri Mondor, Assistance Publique-Hôpitaux de Paris, Créteil, France; 18 INSERM U955, Team «Viruses, Hepatology, Cancer», Créteil, France

**Keywords:** Microbiology, Medical research

## Abstract

We aimed to explore the relationships between specific viral mutations/mutational patterns and ventilator-associated pneumonia (VAP) occurrence in COVID-19 patients admitted in intensive care units between October 1, 2020, and May 30, 2021. Full-length SARS-CoV-2 genomes were sequenced by means of next-generation sequencing. In this prospective multicentre cohort study, 259 patients were included. 222 patients (47%) had been infected with pre-existing ancestral variants, 116 (45%) with variant α, and 21 (8%) with other variants. 153 patients (59%) developed at least one VAP. There was no significant relationship between VAP occurrence and a specific SARS CoV-2 lineage/sublineage or mutational pattern.

## Introduction

Current data on ventilator-associated pneumonia (VAP) complicating COVID-19-associated acute respiratory distress syndrome (ARDS) have been mainly obtained during the first wave of COVID-19^1^. Yet, bacterial superinfection may be affected by the nature of the infecting viral variants and the widespread use of corticosteroids^2^. There is scarce information in the literature on the rate of superinfections in COVID-19 patients, especially of VAP, according to variant types in the dexamethasone era. Potential explanations for the high incidence of VAP in COVID-19 patients include the long duration of invasive mechanical ventilation support, the high incidence of ARDS, and the administration of immune-suppressive treatment. Specific risk factors for VAP, including SARS-CoV-2-related pulmonary lesions and bacteria-virus interaction in lung microbiota, might also play a role in VAP pathogenesis. As the upper respiratory tract microbiome composition significantly changed as COVID-19 severity increased^3^, we hypothesize that specific SARS-CoV-2 mutations, either in the spike protein or in another viral protein, could have an impact on VAP prevalence.

In this study, we aimed to explore the relationships between SARS-CoV-2 variant lineage/sublineage and specific viral mutations/mutational patterns with VAP occurrence in COVID-19 patients in the dexamethasone era.

## Patients and methods

This is a prospective multicentre observational cohort study. Patients admitted between October 1, 2020 (week 40/20) and May 30, 2021 (week 21/21) in one of the 11 participating ICUs from Greater Paris area hospitals included in the ANTICOV study (NCT04733105) were eligible for study inclusion. Inclusion criteria were as follows: age ≥ 18 years, SARS-CoV-2 infection confirmed by a positive reverse transcriptase-polymerase chain reaction (RT-PCR), patient admitted in the ICU who required mechanical ventilation more than 48 h for acute respiratory failure. Patients with a low viral load (PCR cycle threshold (Ct) > 32) in nasopharyngeal samples were not included in the study.

The primary endpoint was the difference in prevalence of the first VAP according to SARS-CoV-2 variants.

### Ventilator associated pneumonia definition

All VAP episodes prospectively diagnosed by attending physicians were evaluated and retained for analyses, provided that they fulfilled the following criteria (the definition of VAP was based on current guidelines^4^): (1) new or progressive persistent pulmonary infiltrates on chest X-ray; combined with (2) purulent tracheal secretions; (3) fever or hypothermia (body temperature ≥ 38.5 °C or ≤ 36.5 °C, respectively) and/or leukocytosis or leukopenia (white blood cells count ≥ 10.4/mL or ≤ 4 ×  10.3/mL, respectively); and (4) a positive quantitative lower respiratory tract sample (endotracheal aspirate [ETA] ≥ 10.5 colony-forming unit [CFU]/mL, bronchoalveolar lavage [BAL] fluid ≥ 10.4 CFU/mL or plugged telescopic catheter [PTC] ≥ 10.3 CFU/mL). Respiratory tract secretions were cultured for VAP diagnosis and susceptibility profiles of recovered microorganisms were recorded. VAP onset was defined as the day on which the lung sample tested positive.

### SARS-CoV-2 genome sequence analysis

Full-length SARS-CoV-2 genomes from all included patients were sequenced by means of next-generation sequencing. Viral RNA was extracted from nasopharyngeal swabs in viral transport medium using NucliSENS^®^ easyMAG kit on EMAG device (bioMérieux, Marcy-l’Étoile, France). Sequencing was performed with the Illumina COVIDSeq Test (Illumina, San Diego, California), that uses 98-target multiplex amplifications along the full SARS-CoV-2 genome. The libraries were sequenced with NextSeq 500/550 High Output Kit v2.5 (75 Cycles) on a NextSeq 500 device (Illumina). The sequences were demultiplexed and assembled as full-length genomes by means of the DRAGEN COVIDSeq Test Pipeline on a local DRAGEN server (Illumina). Lineages and clades were interpreted using Pangolin and NextClade, before being submitted to the GISAID database (https://www.gisaid.org). Phylogeny was performed after full-length genome alignment with Muscle v3.8.31 (maximum-likelihood model GTR + I; 1000 bootstrap replicates), by means of IQ-Tree v1.3.11.1 and iTOL.

### Statistical analysis

We used a competing risk model (cumulative incidence function of the Gray model)^5,6^ to properly estimate the effect of COVID-19 on VAP development, while considering death and ventilator weaning as competing events using cmprsk package developed by Gray in R software ([http://biowww.dfci.harvard.edu/~gray/cmprsk_2.1-4.tar.gz]). Two-sided p values < 0.05 were considered significant.

### Ethical approval and consent to participate

The study was approved by the Comité de Protection des Personnes Nord-Ouest IV (N° EudraCT/ID-RCB: 2020-A03009-30). Informed consent was obtained from all patients or their relatives. All study procedures and processes were conducted in accordance with the Declaration of Helsinki and with the ethical standards of the institutional and/or research committee.

## Results

Between October 1, 2020, and May 30, 2021, 845 patients were admitted in one of the 11 participating ICUs. Among them, 737 had at least one positive SARS-CoV-2 RNA RT-PCR performed in a nasopharyngeal swab sample in the hospital; 413 patients with a cycle threshold (Ct) ≤ 32 and a nasopharyngeal sample that could be used for full-length viral genome sequence analysis were included in this study. Among these 413 patients, 267 were mechanically ventilated more than 48 h. Patients’ characteristics are shown in Table 1. Most patients had ARDS criteria, including 240 (93%) patients who needed prone positioning, and 52 (20%) requiring veno-venous ECMO support. Among these 259 patients, 122 (47%) had been infected with so-called ancestral “pre-existing” variants, *i.e.*, variants circulating before the emergence of variant α, 116 (45%) with variant α (B.1.1.7), and 21 (8%) with other variants, including β (B.1.351) (n = 19, 4.6%), γ (P.1) (n = 2, 0.5%) and other variants of interest (n = 12, 2.9%). One hundred and fifty-three patients (59%) developed at least one VAP episode.

**Table 1:** Characteristics of patients with Coronavirus disease 19 (COVID-19) (n = 259).

Variables	Total (n = 259)
Age—median [IQR]	65 [58–71]
Male gender	185 (71.4%)
Medical history	
Diabetes mellitus	80 (30.9%)
Congestive heart failure	25 (9.7%)
Hypertension	138 (53.3%)
COPD	20 (7.7%)
Chronic renal failure	36 (13.9%)
Dialysis	13 (5.0%)
Liver cirrhosis	7 (2.7%)
Current smoking	31 (12.0%)
Solid cancer	15 (5.8%)
Blood cancer	13 (5.0%)
Organ transplant	15 (5.8%)
HIV infection	4 (1.5%)
Frailty scale—median [IQR]	3 [2–4]
Clinical characteristics upon ICU admission	
SAPS II- median [IQR]	37 [30–48]
Baseline SOFA- median [IQR]	4 [3–8]
ARDS (Berlin definition)	230 (88.8%)
Norepinephrine	67 (25.9%)
Antibiotics	186 (71.8%)
Treatment during stay	
Corticosteroids	242 (93.4%)
Tocilizumab	31 (12.0%)
Neuromuscular blockade	254 (98.1%)
Prone position	240 (92.7%)
Inhaled nitric oxide	26 (10.0%)
Extra-corporeal membrane oxygenation	52 (20.1%)
Death in ICU	115 (44%)

*COPD* chronic obstructive pulmonary disease, *HIV* human immunodeficiency virus, *SAPS II* Simplified Acute Physiology Score II, *SOFA* sequential organ failure assessment, *ARDS* acute respiratory distress syndrome, *ICU* intensive care unit; variables are shown as median [quartile 1-quartile 3] and number (percentage).

**Figure1:**
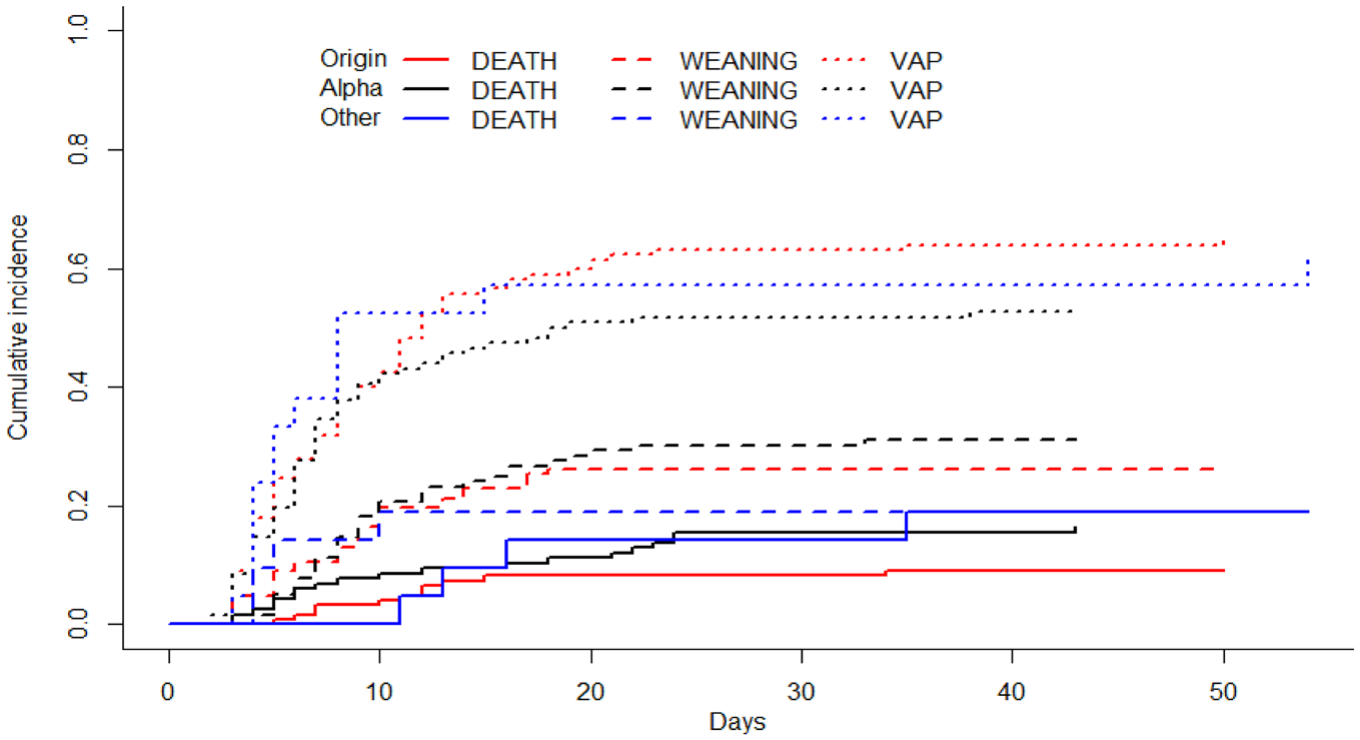
Day-60 cumulative probability of ventilator-associated pneumonia (VAP) in patients infected with the variant of origin (red lines), variant α (black lines) or other variants (blue lines). Cumulative incidence estimated using the Kalbfleish and Prentice method considering time from intubation to VAP (dotted line), to death (continuous line) and to weaning (dashed line).

During the study period, variant α was the dominant VOC in the Greater Paris area. Thus, we first assessed whether patients infected with variant α had a higher risk of developing VAP than others (merging patients infected with preexisting variants and other variants) during the ICU stay. The Fine and Gray model showed that the probability of VAP was not significantly different in the two groups [sub-hazard ratio = 1.26 (0.62–2.54), p = 0.53, Fig. 1] after adjusting for weaning and death as competing events.

Pre-existing and emerging variants (VOC or VOI) are characterized by multiple lineage-specific deletions and amino acid substitutions in their viral genomes. The Spike glycoprotein undergoes evolutive convergence at several amino-acid signature sites found in VOC and VOI circulating at the time of the study period. These sites were selected a priori for in-depth analysis. We found no significant relationship between VAP occurrence and any specific mutation at these sites (Table 2). These results persisted after adjusting for age, sex, antibiotic at admission and SAPS II (Table 3). Although one mutation (A67V) was found to be significantly associated with the risk of VAP (p < 0.05), the number of patients harboring this mutation was considered too small (n = 1) to draw clinically relevant conclusions.

**Table 2:** Association between relevant mutations [(substitutions, deletions (Del)] in spike selected a priori and risk of ventilator-acquired pneumonia by univariable Fine and Gray model analysis.

Mutations	Missing data	VAP		Total	SHR (CI 95%)
		Yes	No		
		N = 153	N = 106	N = 259	
del6970	0	61 (39.9%)	55 (51.9%)	116 (44.8%)	0.77 [0.56; 1.06]
del140145	0	61 (39.9%)	55 (51.9%)	116 (44.8%)	0.77 [0.56; 1.06]
del242244	0	8 (5.2%)	5 (4.7%)	13 (5.0%)	1.12 [0.54; 2.30]
L5F	0	4 (2.6%)	3 (2.8%)	7 (2.7%)	0.89 [0.36; 2.20]
L18F	0	4 (2.6%)	3 (2.8%)	7 (2.7%)	1.04 [0.37; 2.94]
**A67V**	**0**	**1 (0.7%)**	**0 (0.0%)**	**1 (0.4%)**	**5.70 [4.27; 7.60]**
D80AG	0	9 (5.9%)	5 (4.7%)	14 (5.4%)	1.26 [0.62; 2.57]
S98F	0	4 (2.6%)	3 (2.8%)	7 (2.7%)	1.04 [0.36; 3.05]
K417NT	0	10 (6.5%)	5 (4.7%)	15 (5.8%)	1.33 [0.69; 2.58]
N439K	0	0 (0.0%)	0 (0.0%)	0 (0.0%)	–
N450K	0	0 (0.0%)	0 (0.0%)	0 (0.0%)	–
L452R	0	3 (2.0%)	2 (1.9%)	5 (1.9%)	1.08 [0.34; 3.43]
S477N	0	38 (24.8%)	21 (19.8%)	59 (22.8%)	1.12 [0.79; 1.57]
T478K	0	0 (0.0%)	0 (0.0%)	0 (0.0%)	–
E484K	0	11 (7.2%)	5 (4.7%)	16 (6.2%)	1.49 [0.77; 2.88]
N501Y	0	71 (46.4%)	61 (57.5%)	132 (51.0%)	0.80 [0.59; 1.09]
A570D	0	60 (39.2%)	55 (51.9%)	115 (44.4%)	0.76 [0.55; 1.04]
D614G	0	149 (97.4%)	104 (98.1%)	253 (97.7%)	0.85 [0.35; 2.06]
H655Y	0	2 (1.3%)	1 (0.9%)	3 (1.2%)	1.32 [0.32; 5.37]
P681H	0	61 (39.9%)	53 (50.0%)	114 (44.0%)	0.81 [0.59; 1.11]
P681R	0	2 (1.3%)	3 (2.8%)	5 (1.9%)	0.57 [0.15; 2.24]

*SHR* sub-hazard ratio, *CI 95%* 95% confidence interval; variables are shown as median [quartile 1-quartile 3] and number (percentage).

Significant values are in [bold].

**Table 3:** Multivariable Fine and Gray model analysis on risk of ventilator-acquired pneumonia.

Mutations	Missing data	SHR	CI 95%
Variant α	0	0.76	[ 0.54; 1.07 ]
Other SARS-CoV-2 variants ^a^	0	1.07	[ 0.62; 1.87 ]
Age	0	1.02	[ 1.01; 1.04 ]
Male sex	0	1.39	[ 0.98; 1.98 ]
Antibiotic at admission	0	0.84	[ 0.59; 1.18 ]
SAPS II	0	1.00	[ 0.99; 1.01 ]

*SHR* sub-hazard ratio, *CI 95%* 95% confidence interval; ^a^pre-existing variant or other variants.

## Discussion

We previously failed to find a significant relationship between variant lineages, including variant α, pre-existing and other variants, and mortality^7^. The main results of this study show the lack of statistically significant relationship between variant lineages or the presence of any of 17 relevant spike substitutions and/or deletions selected a priori and VAP occurrence. These findings suggest that the occurrence of VAP is not related to the virological nature of the infecting agent, but to non-virological causes.

The full-length SARS-CoV-2 genomes of 413 critically ill COVID-19 patients from 11 ICUs, who were predominantly infected with pre-existing and α (B.1.1.7) variants, were sequenced and the relationship between viral sequences and VAP occurrence was studied. The main results of this study show the lack of statistically significant relationship between variant lineages or the presence of any of 17 relevant spike substitutions and/or deletions selected a priori and VAP occurrence. Previous large-scale data have suggested that patients infected with variant α (B.1.1.7), identified using the SGTF proxy, had a higher risk of dying^8–10^. Yet, no specific comorbidity or clinical conditions was shown to interact with variant types in the previous studies. Interestingly, following SARS-CoV-2-infected patients with different disease severities through the pathway of disease management, Grint et al*.* reported a weakening association between variant α (B.1.1.7) infection and mortality, highest in the primary care population, lower in the hospitalized population, not significant in patients admitted in the ICU^10^. Moreover, in another cohort of 496 hospitalized patients in the United Kingdom, 341 samples positive for SARS-CoV-2 on PCR were sequenced and analyzed, including 198 with B.1.1.7 variant (α) and 143 with non-B.1.1.7 variant infection. No association with variant and death was found with or without adjustment for confounding variables^11^.

Together, our results indicate that, in patients with the most severe forms of COVID-19 who required ICU admission, there was no particular mutational pattern associated with VAP. Therefore, the occurrence of VAP is not related to the virological nature of the infecting agent, but to other causes.

### Limitations and strengths of the study

We acknowledge that our study has some limitations. In this cohort, included patients were predominantly infected with pre-existing and α (B.1.1.7) variants, while the inclusion period ended before the emergence of the δ and ο variants, precluding the generalizability of our findings to these more recent variants. The number of patients included was limited and thus the statistical power may have been too weak to show between-group differences. Yet, there was no clear trend regarding associations between variant groups/mutations and VAP, suggesting that increasing the number of patients in the cohort would not have changed the results.

Our study also has strengths, including the constitution of a prospective multicenter cohort of well-phenotyped critically ill patients, and the fact that we performed full-length SARS-CoV-2 genome sequencing in a large number of patients. The start of inclusion of the present study was October 1, 2020, which corresponded in France to the beginning of the second COVID-19 wave, a period when ICU admission policies, ICU patient load and demand, and COVID-19 management strategies were more homogeneous among centres than during the first COVID-19 wave. The high incidence of VAP described in our study is in line with that reported in previous studies^1,12^.

## Conclusions

We found no association between the variant status or any mutational pattern in SARS CoV-2 viral genes and VAP, indicating that the occurrence of VAP is related to non-virological causes.

## Data Availability

The datasets supporting the conclusions are included within the article. The datasets used and/or analysed during the current study are available from the corresponding author on reasonable request.

## Abbreviations

ARDS: Acute respiratory distress syndrome
ICU: Intensive care unit
ECMO: Extra-corporeal membrane oxygenation
VOC: Variant of concern
VOI: Variant of interest
VAP: Ventilator associated pneumonia
COVID 19: Coronavirus disease 2019
